# Giant Colonic Lipoma Presenting as Intermittent Colonic Obstruction With Hematochezia

**DOI:** 10.7759/cureus.11434

**Published:** 2020-11-11

**Authors:** Anabel Liyen Cartelle, Pearl Princess Uy, John Erikson L Yap

**Affiliations:** 1 Division of Gastroenterology and Hepatology, Medical College of Georgia at Augusta University, Augusta, USA

**Keywords:** endoscopic approach, hematochezia, bowel obstruction, giant colonic lipoma

## Abstract

Colonic lipomas are rare benign, non-epithelial tumors of mesenchymal origin. They are often solitary lesions of submucosal origin found in the proximal colon and typically measure less than 2 cm in size. Giant colonic lipomas are greater than 4 cm and present with non-specific gastrointestinal symptoms such as abdominal pain, abdominal distention, constipation, or gastrointestinal bleeding. Traditionally, giant colonic lipomas have been surgically rather than endoscopically resected due to concerns for bowel wall perforation and life-threatening hemorrhage. However, in recent years, advances in endoscopic tools and hemostatic techniques have lessened these risks. The following case details the successful endoscopic resection of an intermittently obstructing giant colonic lipoma (6 cm) located in the descending colon utilizing the loop-assisted-snare resection technique.

## Introduction

Intestinal lipomas are rare benign, non-epithelial tumors of mesenchymal origin that can form throughout the gastrointestinal tract. They are more frequently encountered in the colon with an overall incidence of 0.3-4.4% [1]. These lesions typically manifest within the ascending colon (45%), followed by the sigmoid colon (30%), descending colon (15%), and transverse colon (9%) [2]. Patients are generally asymptomatic as most of these lesions typically measure less than 2 cm in size. However, those with giant lipomas (>4 cm) almost always present with non-specific gastrointestinal symptoms such as abdominal pain, abdominal distention, constipation, or gastrointestinal bleeding [3]. Colonic lipomas can be detected using either endoscopic or radiological modalities such as computed tomography (CT) or magnetic resonance imaging (MRI). Histologically, these lesions are characterized by well-circumscribed mature adipose tissue originating in the submucosa, with overlying mucosa that may be normal or contain an adenoma or a serrated polyp [4]. Traditionally, giant colonic lipomas were surgically rather than endoscopically resected due to concerns for bowel wall perforation [5]. However, in the recent years, advances in endoscopic tools and hemostatic techniques have lessened this risk. We present the case of a middle-aged female presenting with symptoms consistent with an intermittent colonic obstruction and concomitant lower gastrointestinal bleeding found to be secondary to a giant colonic lipoma that was subsequently successfully treated via complete endoscopic resection.

## Case presentation

**Figure 1:**
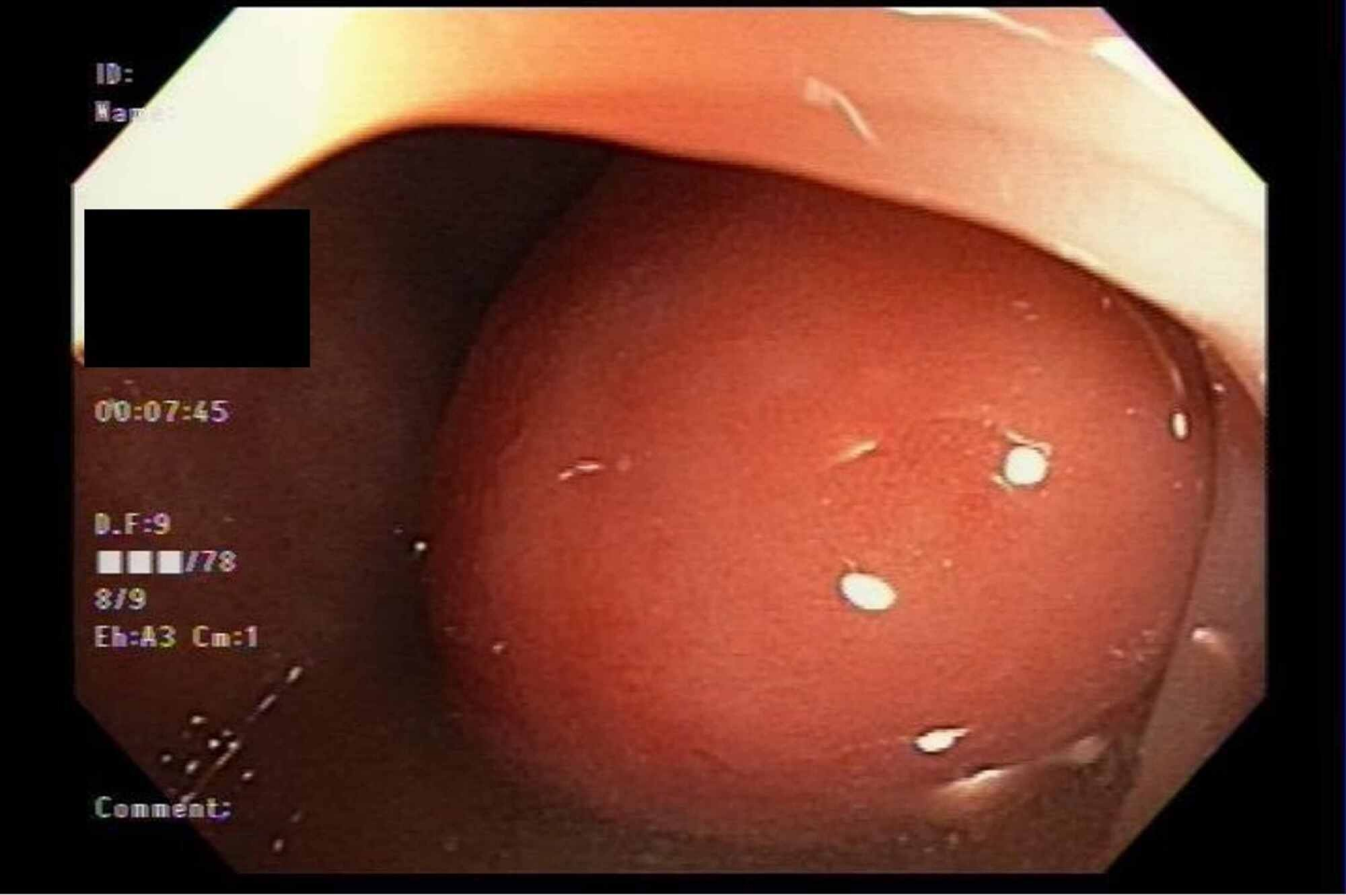
Colonoscopy revealed a large 6 cm pedunculated polypoid lesion in the descending colon causing a ball-valve type intermittent colonic obstruction of the lumen

A 40-year-old African American female with a past medical history of hypertension and lupus profundus was referred to the gastroenterology clinic by her primary physician for endoscopic evaluation of a one-year history of intermittent left lower quadrant pain, constipation, and hematochezia. The patient described noticing a bulge located in her left lower quadrant when symptomatic that would subsequently disappear after having a bowel movement. She often needed to massage the area and change position to facilitate a bowel movement. Upon further questioning, the patient denied use of nonsteroidal anti-inflammatory drugs (NSAIDs), opiates or other medications known to cause constipation. She denied any personal or family history of colon cancer. Her physical exam, including a digital rectal exam, was unremarkable. Laboratory tests including hemoglobin and hematocrit were also within normal range. A colonoscopy was performed which revealed a large broad-based 6 cm pedunculated polypoid lesion at the descending colon that appeared to be causing a ball-valve type intermittent colonic obstruction (Figure 1).

**Figure 2:**
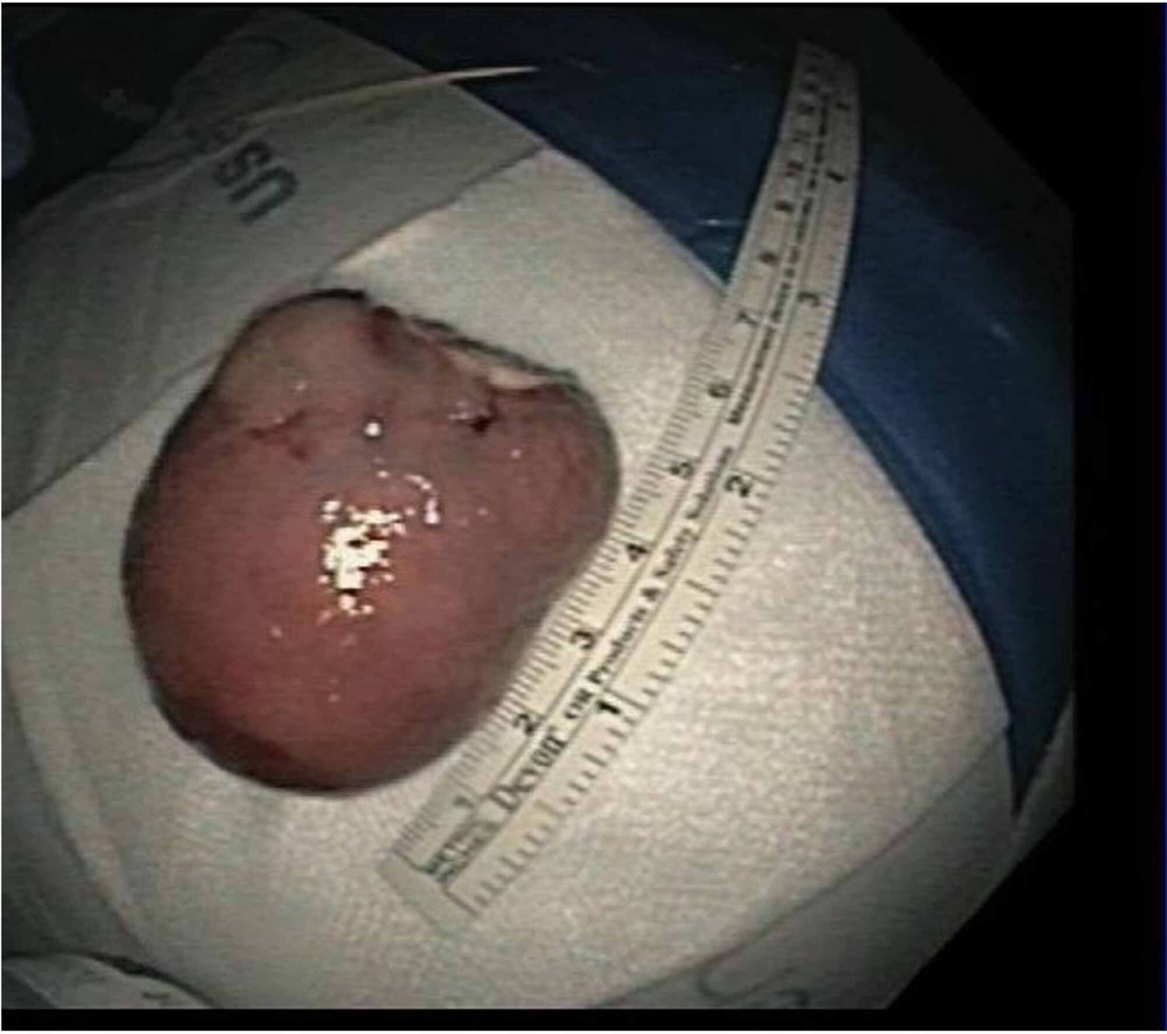
An endoloop was deployed at the stalk and the entire polypoid lesion was resected en bloc with snare cautery

Grossly, the mass had normal overlying mucosa with evidence of slight erosions at the tip. Using biopsy forceps, the polypoid lesion was investigated and demonstrated a positive pillow sign. An endoloop device was then deployed at the stalk and the entire mass was endoscopically resected with snare cautery en-bloc and removed from the colon using a net retrieval device (Figure 2).

**Figure 3:**
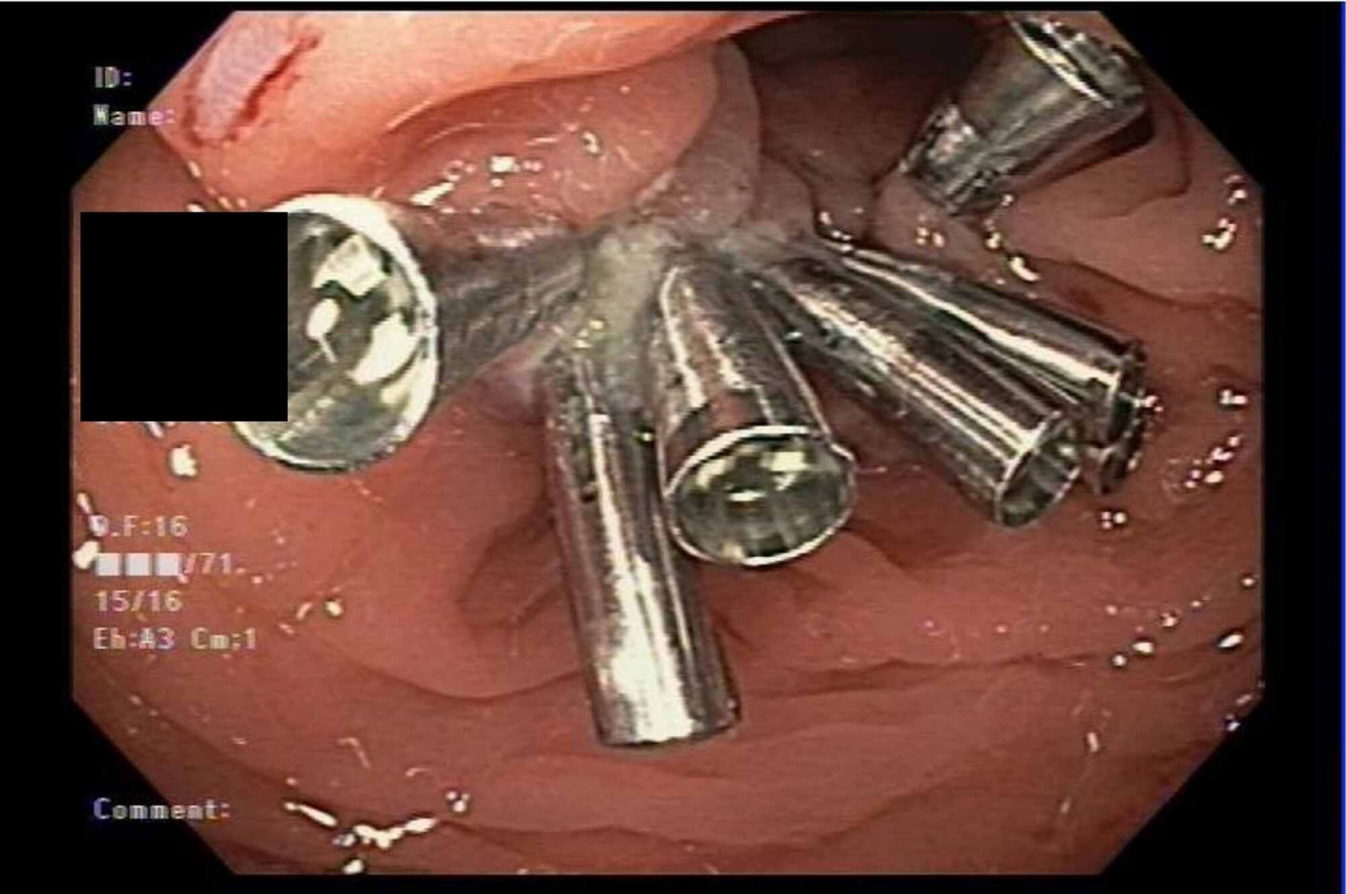
A small, colonic bowel wall defect was detected on inspection of the snare cautery site and completely closed using seven hemoclips

Upon inspection of the snare cautery site, the endoloop was noted to have been inadvertently cut and a small, colonic bowel wall defect was noted. The defect was completely closed utilizing seven hemoclips (Figure 3).

Pathology of the large polypoid mass revealed colonic mucosa with underlying mature adipose tissue consistent with a submucosal colonic lipoma. The patient reported complete resolution of all her symptoms upon follow-up.

## Discussion

Colonic lipomas are uncommon benign tumors composed of mature adipose tissue. They are often solitary lesions of submucosal origin, and very rarely of subserosal and muscularis origin [6]. Most are less than 2 cm in size but have been reported in the literature to grow up to 30 cm [7]. They are detected predominantly in women between the ages of 50-65 years via colonoscopy, surgery, or autopsy [8]. Individuals with lesions less than 2 cm in size are typically asymptomatic. For larger lesions that have significant mass effect, presenting symptoms can include abdominal pain, change in bowel habits, and rarely, gastrointestinal bleeding, perforation, or intestinal obstruction which can also raise suspicion for malignancy. Giant lipomas in specific have been linked with cases of intussusception, wherein one segment of the intestine invaginates or telescopes inside another adjoining intestinal segment, a rare clinical condition in adults [6]. Our patient’s transient constipation, abdominal pain, and left lower quadrant bulge were indicative of an intermittent colonic obstruction secondary to a giant lipoma via the ball-valve type effect. 

Given the nonspecific nature of the gastrointestinal symptoms related to colonic lipomas, its clinical diagnosis can be very challenging and requires additional diagnostic radiological or endoscopic modalities. An abdominal CT scan can demonstrate the lesion’s characteristic uniform low-density adipose tissue signal with any overlying hyper-intensity stranding near the mucosal surface indicating the presence of ulcerations [9]. Endoscopically, there are three classic signs characteristic of colonic lipomas: 1) the tent-sign: tent-like appearance of the mucosa when its grabbed over the lesion and pulled away; 2) the pillow sign: depression or pillowing of mass when forceps are pressed into it; and 3) the naked fat sign: extrusion of fat tissue after biopsy [10].

Traditionally, endoscopic removal of lipomas had been reserved for smaller lesions (<3 cm) due to concerns of increased risk of bowel wall perforation and life-threatening hemorrhage. However, in recent years, advances in endoscopic procedures and hemostatic tools such as hemoclips, endoloops, and hemostatic powders have gained it favor over more invasive surgeries [11]. Depending on the lipoma’s morphology, pedunculated vs flat, there are several endoscopic approaches to treatment. For pedunculated lesions, endoscopic mucosal resection (EMR), “loop and let go”, loop-assisted-snare resection, and endoscopic submucosal dissection (ESD) are favored. EMR was the first technique used to endoscopically remove colonic lipomas [12]. It involves the injection of the base of a lesion for submucosal lift and the use of a standard cautery snare for resection. It is especially favored in lipomas with narrow, elongated stalks and has a demonstrated high rate of definitive resection. However, EMR is also associated with a higher risk of bleeding given the poor electrical conductivity of cautery through adipose tissue [13]. “Loop and let go” is a newer technique that utilizes an endoloop at the base of the lipoma to achieve spontaneous resection via secondary necrosis. A recent prospective validation study by Ivekovic et al. [14] confirmed the safety and efficacy of this method for large colonic lipomas, highlighting its advantage of forgoing electrocautery all together. The loop-assisted-snare resection method, utilized in our case, combines the two previous techniques by utilizing the endoloop to snare the base of the lipoma and then removing it using the cauterized snare. Also highlighted in our case is one of the major pitfalls with this technique, the failure of the endoloop at the base in the instance of a broad stalk. Fortunately for us, we were able to control bleeding and successfully close the small, colonic bowel wall perforation with hemoclips. Lastly, ESD utilizes different electrosurgical tools to expose the submucosal layer of the colonic tissue and allows for the precise excision of the lesion. However, in more resource depleted regions, the tools necessary for this technique and access to providers experienced enough to perform it remain the main limiting factors. For flat/sessile lesions, a somewhat less effective resection method can be utilized, the unroofing technique, which involves the excision of the superior half of the lipoma via polypectomy snare [12].

Currently, there is limited literature comparing the efficacy and safety of these various endoscopic techniques for the resection of colonic lipomas, highlighting their relative symptomatic rarity. The most comprehensive systematic review available was done by Bronswijk et al. [15], encompassing 24 different studies, half of which reported an average lesion size of 5 cm or greater, and totaling 77 giant colonic lipomas. The techniques evaluated in this review included unroofing, ESD, EMR and loop-assisted-snare resection. Of these, EMR and loop-assisted resection demonstrated superior endoscopic remission rates at the cost of increased adverse events, 12.9% and 13.8%, respectively, compared to none in the unroofing and ESD group. However, the reliability of the conclusions of this review were severely limited by the small sample size and the high variability in approach to each endoscopic technique between individual studies. As such, per the review's conclusions, until higher quality, larger scale studies are available, the mode of endoscopic resection will ultimately depend on practitioner experience and individual patient characteristics. As opposed to its predominance in years past, the role of surgery is now reserved for cases involving very large sessile lesions that are difficult to ensnare. Additionally, lipomas arising from the serosal or muscularis layers are also at higher risk of complications and thus are generally still treated surgically [16]. 

## Conclusions

Previously, surgical resection was recommended for the management of giant colonic lipomas due to a higher risk of bowel wall perforation and life-threatening hemorrhage with endoscopic resection. However, endoscopic techniques such as unroofing, endoscopic mucosal resection (EMR), “loop and let go”, loop-assisted-snare resection, and endoscopic submucosal dissection (ESD) have been effectively used in recent years to excise giant colonic lipomas. This case highlights the endoscopic resection of an obstructing giant descending colonic lipoma utilizing loop-assisted-snare resection technique while also addressing possible intraprocedural complications including endoloop failure and small, colonic bowel wall perforation.
